# Serological investigation of SARS-CoV-2 infection in patients with suspect measles, 2017–2022

**DOI:** 10.1186/s12985-023-02117-9

**Published:** 2023-07-20

**Authors:** Silvia Bianchi, Clara Fappani, Maria Gori, Marta Canuti, Daniela Colzani, Maria Cristina Monti, Camilla Torriani, Mario C. Raviglione, Gianvincenzo Zuccotti, Elisabetta Tanzi, Antonella Amendola

**Affiliations:** 1 Department of Health Sciences, Universita’ degli Studi di Milano, Milan, Italy; 2 Coordinated Research Center “EpiSoMI”, Universita’ degli Studi di Milano, Milan, Italy; 3 Department of Clinical Sciences and Community Health, Milan, Italy; 4 Department of Pathophysiology and Transplantation, Universita’ degli Studi di Milano, Milan, Italy; 5 Centre for Multidisciplinary Research in Health Science (MACH), Universita’ degli Studi di Milano, Milan, Italy; 6 Unit of Biostatistics and Clinical Epidemiology, Department of Public Health, Experimental and Forensic Medicine, University of Pavia, Pavia, Italy; 7 Department of Paediatrics, Children Hospital V. Buzzi, Universita’ degli Studi di Milano, Milan, Italy; 8 Romeo and Enrica Invernizzi Pediatric Research Center, Universita’ degi Studi di Milano, Milan, Italy

**Keywords:** SARS-CoV-2, COVID-19, Pre-pandemic, Measles surveillance, Virus emergence, Rash

## Abstract

**Background:**

Several studies suggested that SARS-CoV-2 was already spreading worldwide during the last months of 2019 before the first outbreak was detected in Wuhan, China. Lombardy (Northern Italy) was the first European region with sustained SARS-CoV-2 transmission and recent investigations detected SARS-CoV-2-RNA-positive patients in Lombardy since late 2019.

**Methods:**

We tested for anti-SARS-CoV-2 IgG all serum samples available in our laboratory (N = 235, collected between March 2017 and March 2022) that we received within the framework of measles/rubella surveillance from measles and rubella virus-negative patients.

**Results:**

Thirteen of 235 samples (5.5%) were IgG-positive. The positivity rate increased starting in 2019 and was significantly different from the expected false positive rate from 2019 onwards. Additionally, in 2019 the percentage of IgG-positive patients was significantly lower among SARS-CoV-2 RNA-negative patients (3/92) compared to SARS-CoV-2 RNA-positive patients (2/7, p = 0.04). The highest percentage of IgG positivity in the pre-pandemic period was recorded during the second half of 2019. This coincided with an increase in negativity for measles and a widening of the peak of the number of measles discarded cases per 100,000 inhabitants, indicating a higher-than-normal number of measles-negative patients experiencing fever and rash. This also coincided with the first patient positive for SARS-CoV-2 RNA (September 12th, 2019); this patient was also positive for anti-SARS-CoV-2 IgG and IgM.

**Conclusions:**

Although the number of samples was low and one cannot conclusively establish that the virus started circulating in Lombardy around September 2019, our findings should stimulate similar research investigating the possibility of undetected SARS-CoV-2 pre-pandemic circulation.

**Supplementary Information:**

The online version contains supplementary material available at 10.1186/s12985-023-02117-9.

## Introduction

Lombardy (Northern Italy) was the Italian region with the highest COVID-19 clinical burden in early 2020 [1], one of the first non-Asian areas that experienced sustained SARS-CoV-2 transmission, and the first epicenter of the European epidemic [2]. Previous molecular and serological investigations showed that likely the virus was already present in Lombardy since late 2019 [3–5]. In our previous investigation, we observed that SARS-CoV-2 RNA could be detected in oropharyngeal swabs and urine collected from patients with morbilliform eruptions that tested negative for measles and rubella viruses starting in September 2019 [5]. Indeed, COVID-19 is associated with skin manifestations. These are highly polymorphic, but morbilliform rash is the most commonly described pattern, and can appear at any stage of the disease, even in the absence of respiratory symptoms [6].

As World Health Organization (WHO)-accredited Subnational Reference Laboratory for measles and rubella surveillance in Lombardy (Network of Italian Reference Laboratories for Measles and Rubella: MoRoNet) we regularly receive samples from patients with fever and rash for molecular and serological diagnosis of measles and rubella [7]. Part of our efforts are also devoted to better investigate these cases by serologically and molecularly characterizing both measles-positive and measles-negative cases [5, 7, 8]. Additionally, the discarded rate (number of non-measles/non-rubella cases in a year divided by the average population in the study area) is measured annually. This is the only indicator of surveillance activity performance. We noticed that the discarded rate was higher in 2019, while the percentage of measles-negative cases increased. This increase coincided with the first molecular detection of SARS-CoV-2 in Lombardy [5].

In the same study, we also evaluated whether SARS-CoV-2 infections could be detected in the same patients by identifying antibodies directed against this virus through ELISA (enzyme-linked immunosorbent assay) [5]. While several samples tested positive, the presence of potential cross-reaction with antibodies against other agents (false positive) complicated data interpretation. Similarly, other investigators also reported the identification of anti-SARS-CoV-2 antibodies in Europe in the fall of 2019 [3, 4, 9], but these studies did not include samples collected before September 2019, making it impossible to compare seropositivity in different years and identify whether an increase in seroprevalence could be observed in 2019. In this study, we expand on our previous investigation, which considered only samples collected from August 2018, and describe the results of anti-SARS-CoV-2 IgG (immunoglobulins type G) ELISA testing performed on all samples collected from patients referred for measles or rubella who tested negative for measles and rubella viruses since March 2017, the beginning of the surveillance activities, until March 2022.

## Methods

The study included all available serum samples (N = 235) collected within the framework of measles/rubella surveillance from measles and rubella negative patients between March 2017 and March 2022. Patients were 112 females (47.7%) and 123 males (52.3%), the median age was 22 (range 0–95 years), and samples were collected 0–23 days (median: 2, IQR: 1–4 days) after rash onset.

Sera were retrospectively tested for anti-SARS-CoV-2 IgG using the semi-quantitative Anti-SARS-CoV-2 IgG ELISA (Euroimmun, Lübeck, Germany) test according to manufacturer’s instructions. Positive samples were those with a sample extinction to calibrator extinction ratio $\ge$1.1.

Numeric variables were reported as the median (IQR). Proportions were reported in percentages for dichotomous count variables, excluding borderline samples (ratio 0.8–1.1). Observed proportions were compared to the expected proportions of false positive results (calculated according to 99.6% of specificity provided by the kit manufacturers), using the test on the equality of proportions and computed Clopper-Pearson 95% confidence intervals. Other proportions were compared with Fisher’s exact tests and, in all cases, two-sided p values < 0.05 were considered statistically significant. A logistic regression analysis (*logit* function) was performed to identify independent variables predictors of dependent variable positivity (IgG+). Statistical analyses were performed using Stata version 17 (StataCorp).

## Results

**Fig. 1:**
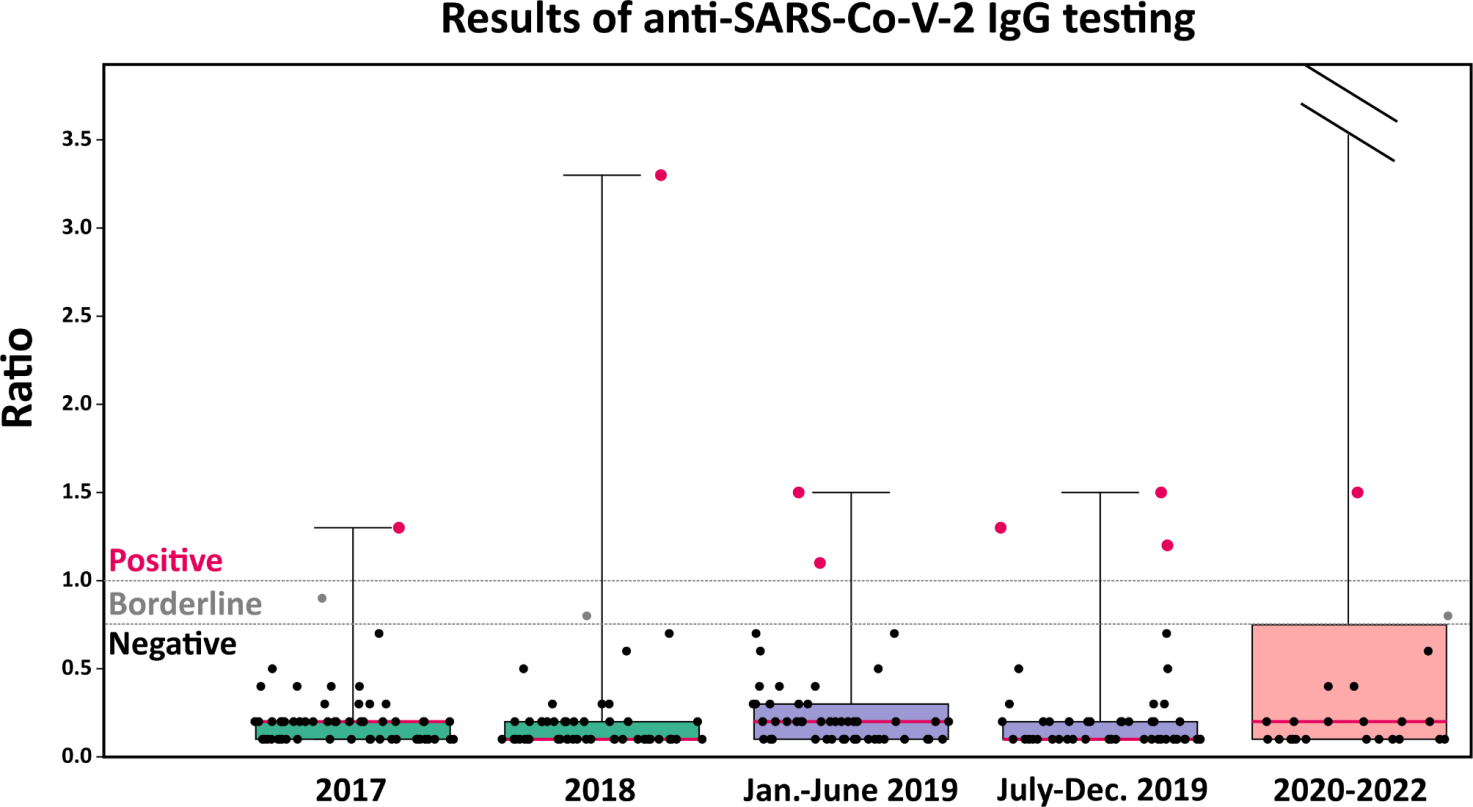
Results of the semi-quantitative anti-SARS-CoV-2 IgG ELISA. Results are displayed as ratio of the extinction of the sample over the extinction of the calibrator and values equal to or above 1.1 were considered positive. For each box-and-whisker plot, the boxes indicate the interquartile range (25th and 75th percentile) with median values displayed as thick red lines, whiskers show the maximum and minimum observed values, and each dot corresponds to one measurement. Results for five positive samples from 2021–2022 are not displayed as out of range (7–10)

Thirteen out of the 235 tested samples (5.5%) were IgG-positive and three (1.3%) were borderline. Figure 1 shows the distribution of IgG measurements and how they were classified (positives, borderline, negatives) by period of serum collection. Signal strength varied between samples, with the strongest values (> 6) documented in 2021 and 2022. Table 1 shows the number of tested and positive subjects per period and the relative proportions of positivity (IgG positivity rate). The observed positive rate increased from 2019 and was significantly different from the expected false positive rate from 2019 onwards. This was also true when analyzing the first and second half of 2019 separately.

**Table 1:** Anti SARS-CoV-2 IgG positive sera collected from patients with fever and rash in different periods of time

Period	N. sample with determined IgG	N. samples IgG-positive and IgG positivity rate (%)	IgG positivity rate 95%CI	p
March - Dec 2017^I,II^	57	1 (1.75%)	0.04–9.39%	0.105
Jan - Dec 2018 ^I,II^	49	1 (2.04%)	0.05–10.85%	0.068
Jan - Dec 2019 ^I^	99	5 (5.05%)	1.66–11.39%	< 0.001*
Jan - June 2019 ^II^	49	2 (4.08%)	0.50-13.98%	< 0.001*
July – Dec 2019 ^II^	50	3 (6.00%)	1.26–16.55%	< 0.001*
Jan 2020 - March 2022 ^I,II^	27	6 (22.22%)	8.62–42.26%	< 0.001*

An * indicates statistically significant differences between the obtained proportions and the expected proportion of false positives

^I^Analysis performed considering 2019 as a whole; ^II^Analysis performed splitting 2019 into two halves

**Fig. 2:**
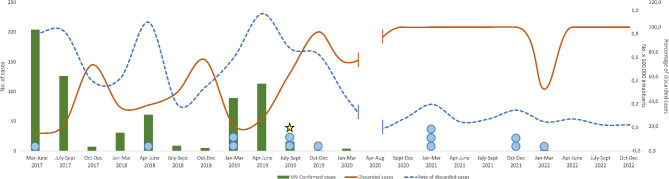
Indicators of measles and rubella surveillance performance and molecular and serological SARS-CoV-2 positivity for the investigated population. The brown continuous line (scale on outer right) indicates the percentage of suspected cases that tested negative for measles and rubella virus throughout the years, the blue dotted line shows the number of discarded cases per 100,000 inhabitants for the same periods (scale on the inner right), while the green bars represent the number of measles positive cases (scale on the left). Data for March-September 2020 are missing as surveillance activities were temporarily interrupted because of the COVID-19 pandemic. Patients that tested positive for anti-SARS-CoV-2 IgG are indicated by blue dots, while the first sample detected to be SARS-CoV-2 RNA-positive is indicated with a star

During the years 2017–2018, the number of measles/rubella discarded cases per 100,000 surveilled inhabitants fluctuated and the percentage of measles/rubella negative suspected cases varied depending to measles epidemic trends (Fig. 2). However, in summer 2019, after a large measles outbreak (last positive detected on July 26, 2019 [7]), the percentage of measles and rubella negative samples abruptly increased (up to 100%). This corresponded also to a widening of the peak of the number of measles/rubella discarded cases per 100,000 inhabitants (normally high during times of high measles circulation) and coincided both with the first molecular identification of SARS-CoV-2 (September 12th, 2019, [5]) and with an increase in anti-SARS-CoV-2 IgG detection rates (Table 1).

Combining our data with previously obtained results [5], the proportion of patients positive for IgG was significantly lower among SARS-CoV-2 RNA-negative patients in 2019 (3/92) compared to SARS-CoV-2 RNA-positive patients (2/7, p = 0.04). One IgG positive patient was also IgM positive (January 2021), one was also positive for viral RNA (October 2019) and, notably, the patient whose samples were collected on September 12, 2019, was positive for IgG, IgM and viral RNA.

The trend analysis did not show a significant temporal trend (Supplementary Figure S1) and sex, age and time between exanthem onset and sample collection were not identified as significant predictors of positivity.

## Discussion

Several studies investigated the pre-pandemic circulation of SARS-CoV-2 and concluded that the virus was likely already circulating outside of China before the first outbreak observed in Wuhan [3]. While PCR-based tests allow to directly detect the virus, false positive results can be obtained with serological assays as cross-reacting antibodies can be present in sera of uninfected patients. Therefore, large-scale investigations analyzing samples collected for some time before COVID-19 emergence would be required to observe a rise in positivity rates consistent with initial viral circulation. Unfortunately, such investigations are still lacking.

We tested for anti-SARS-CoV-2 IgG all sera received for measles/rubella surveillance collected from measles and rubella virus-negative patients. Indeed, patients with COVID-19 can develop morbilliform rash [6, 10, 11] and our previous investigations demonstrated that both SARS-CoV-2 RNA and antibodies against this virus can be detected in these patients [5]. IgG were chosen because the available detection assay was characterized by a high specificity (99.6%). Additionally, not all patients produce IgM, and their level decreases a few days after symptom onset. Although there is individual variation, IgG against SARS-CoV-2 develop ~ 2 weeks after infection, when the virus may no longer be detectable, and remain measurable for a long time [12]. Similarly, cutaneous manifestations appear later during SARS-CoV-2 infection, up to 4 weeks after the onset of respiratory symptoms [10, 11], with a timeline more consistent with IgG levels.

Thirteen patients were positive for anti-SARS-CoV-2 IgG and seven of them were symptomatic during the pre-pandemic period. Given the low number of investigated samples and identified positives, we compared the percentage of detected positive samples to the expected proportion of false positive results (0.04%) and observed that the difference between these two proportions was statistically significant for the year 2019, including when considering the first and second half separately, but not for the years 2017 and 2018. Additionally, in 2019, the percentage of IgG-positive patients was significantly lower among SARS-CoV-2 RNA-negative patients than among SARS-CoV-2 RNA-positive patients.

The semi-quantitative method we used showed that the strength of the signal increased in 2021–2022. Although weakly reacting antibodies could indicate a false positive signal, highly reacting antibodies identified in a few patients symptomatic in later phases of the pandemic (2021–2022) could have been determined by re-exposures or vaccination. Indeed, three of the five strongly positive samples were collected after COVID-19 mass vaccination campaigns had started and all five were sampled after at least one year of virus circulation.

The highest percentage of IgG positivity during the pre-pandemic period was during the second half of 2019, with three positive samples in September and October. This coincided with an increase in negativity for measles and a widening of the peak of the number of discarded cases per 100,000 inhabitants, which followed a measles epidemic that ended in July. In other words, while measles virus stopped circulating, there was still a higher-than-normal number of patients experiencing fever and rash. This is consistent with the observed increase in measles discarded rate for the year 2019 [5]. Noticeably, this is also the time when we detected the first SARS-CoV-2 RNA-positive sample (September 12th, 2019, [5]), and it may be that this is the moment when SARS-CoV-2 started circulating in Lombardy. These results are in agreement with other studies identifying IgG and neutralizing antibodies against SARS-CoV-2 in the same time period in Europe [3, 4, 9].

This study has some limitations. Firstly, the low number of available samples limits our findings and the power of our statistical analyses. Secondly, the investigated period is limited to 3 pre-pandemic years and 3 pandemic years with a variable number of samples in each period. However, the population type was consistent across the years of the study. Additionally, confirmation assays, such as neutralization, was not performed and, while the specificity of the used assay is high, the kit is characterized by a low sensitivity (47.3% and 94.4% before and after day 10 after symptom onset, respectively) and some cases may have gone undetected. Nonetheless, although this study cannot conclusively establish when the virus started circulating in Lombardy, it should serve the purpose of stimulating further research as evidence for undetected SARS-CoV-2 pre-pandemic circulation continues to accumulate.

### Electronic supplementary material

Below is the link to the electronic supplementary material.

Supplementary Material 1

## Data Availability

The data that support the findings of this study are available from the corresponding author upon reasonable request.
