# Supplementary material for: Serological investigation of SARS-CoV-2 infection in patients with suspect measles, 2017–2022
Source: Virol J. 2023 Jul 20;20:160. doi: 10.1186/s12985-023-02117-9 (PMC10357797; doi:10.1186/s12985-023-02117-9)

**Serological investigation of SARS-CoV-2 infection in patients with suspect measles, 2017-2022**

Silvia Bianchi, Clara Fappani, Maria Gori, Marta Canuti, Daniela Colzani, Maria Cristina Monti, Camilla Torriani, Mario Raviglione, Gianvincenzo Zuccotti, Elisabetta Tanzi, Antonella Amendola

**Supplementary Figure S1. Trend of IgG proportion throughout the investigated period.** The analysis was performed both considering 2019 as a whole or split into two halves (insert). Dots represent identified frequencies, while bars correspond to 95% IC (Clopper-Pearson).


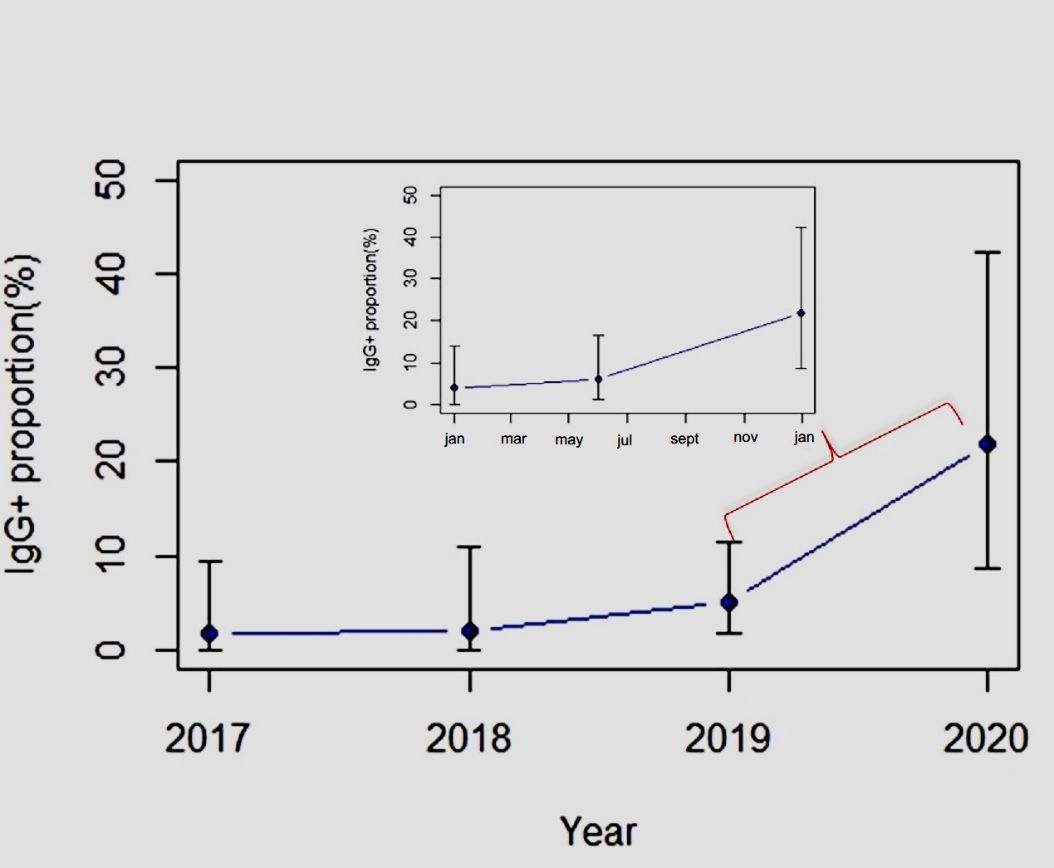

Supplement: Supplementary file 1 — Supplementary Material 1 [file 12985_2023_2117_MOESM1_ESM.docx]
